# Manganese Enhances Prion Protein Survival in Model Soils and Increases Prion Infectivity to Cells

**DOI:** 10.1371/journal.pone.0007518

**Published:** 2009-10-21

**Authors:** Paul Davies, David R. Brown

**Affiliations:** Department of Biology and Biochemistry, University of Bath, Bath, United Kingdom; University of Nebraska Medical Center, United States of America

## Abstract

Prion diseases are considered to be transmissible. The existence of sporadic forms of prion diseases such as scrapie implies an environmental source for the infectious agent. This would suggest that under certain conditions the prion protein, the accepted agent of transmission, can survive in the environment. We have developed a novel technique to extract the prion protein from soil matrices. Previous studies have suggested that environmental manganese is a possible risk factor for prion diseases. We have shown that exposure to manganese is a soil matrix causes a dramatic increase in prion protein survival (∼10 fold) over a two year period. We have also shown that manganese increases infectivity of mouse passaged scrapie to culture cells by 2 logs. These results clearly verify that manganese is a risk factor for both the survival of the infectious agent in the environment and its transmissibility.

## Introduction

Prion diseases or transmissible spongiform encephalopathies (TSE) remains a major problem in several countries despite concerted efforts to eradicate the disease. As the pattern of incidence rules out spontaneous outbreak of disease, evidence suggests the environment is the source of the infective agent [1], [2], [3]. This agent may enter the soil via infected carcasses, meat products or farm effluent [4]. It has been shown that some residual infectivity remains after three years in soil that had been exposed to infected material [5]. Natural processes in the soil such a bacterial activity, exposure to UV radiation and soil acidity should be deleterious for even the most resilient organic material.

The infective agent in prion diseases is now accepted to be an abnormal isoform of the prion protein (PrP) [6]. Therefore, in order for the infective agent to survive in the environment, significant amounts of PrP must be able to resist normal mechanisms of protein degradation and it is currently not known how this would be possible. PrP is a metal binding protein with high affinity for copper [7], [8] but also has affinity for manganese similar to other manganese binding proteins [9]. Interactions with metals may be able to contribute to the proteins stability and resistance against degradation in soil. For example, it has been shown that manganese can cause PrP to fold into a proteinase resistant form [10]. Certainly, many metals exist within soils and it may therefore be possible that these interactions contribute to PrP's longevity in the environment. There is also some evidence of high manganese and low copper levels in areas of high scrapie incidence [11].

A clear understanding of how PrP interacts with soils is important in order to assess whether metals contribute to the stability of PrP in the environment.

The mechanism of protein adsorption on to soil particles is far from straight forward. Complicating factors include soil pH and constituents and protein PI, conformation, size, charge, solubility and flexibility [12], [13]. The majority of soil/protein interactions have, therefore, been studied with model systems, especially with constituent clays such as kaolinite (kte) and montmorillonite (mte). One study [14] showed the importance of electronegative interactions in the adsorbance of bovine serum albumin (BSA) onto mte and how the strength of these interactions could alter the conformation and properties of the protein. Other studies have shown specifically the very strong adsorptive nature of soil clays to proteins, especially prions. Leita et al [1] demonstrated the difficulty in desorbing prions from clay, especially mte and suggested that the conditions in most soils would favour an accumulation of stable prions in soils exposed to contaminated material. Another such study suggested that mte would promote an orientation of PrP towards the soil involving elements across the entire protein in both the N and C terminus, making the adsorption almost irreversible in its strength [15]. Another study confirmed that PrP could adsorb strongly to clay and remain infectious [16]. With the protein/soil interactions well characterised, it is possible to construct model systems whereby the stability of both Metal bound and apo forms of PrP^c^ and PrP^sc^ can be studied.

The connection between prion disease and metals is well established. In particular, there is evidence that patients and animals with TSEs have altered level of manganese often in specific regions associated with the loss of neurons [17], [18], [19], [20], [21]. Cells infected with prions show elevated levels of manganese and also show increased expression of the protein DMT-1 associated with uptake of manganese into cells [22]. Altering the diet to increase manganese results in an increased expression of PrP in the brain [22] and increased expression of PrP is known to increase the chance of prion infection [23]. There is already some evidence that altered copper levels modulate prion infection in animal models of TSE but it is still unclear as to whether manganese changes in the brain are secondary to prion infection or whether the increase manganese increases susceptibility to infection.

The aims of the study were to assess the stability of recombinant PrP on a model soil over a period of 24 months in the presence or absence of copper and manganese metals. The stability of PrP^c^ and PrP^sc^ in the presence of soils and metals was also studied. We also examine the ability of manganese to modulate infection of cultured cells with scrapie. Our results demonstrate that manganese dramatically increases PrP survival in soils and lowers the effective dose needed to infect cells with scrapie.

## Results

### Adsorption properties of model soils

**Figure 1:**
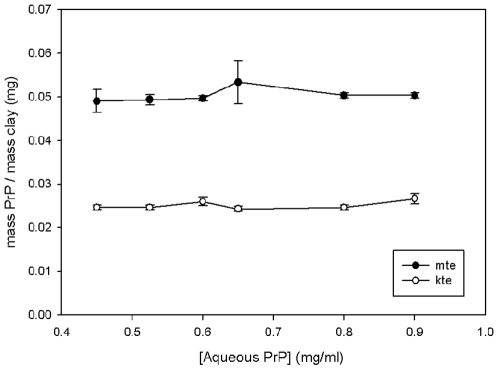
Adsorpative capacity of mte and kte for rPrP. The equilibrium of adsorbed PrP to clay for kte and mte over a range of aqueous protein concentrations from 0.4 to 1 mg/ml.

Initially, an evaluation of the total capacity of mte and kte for PrP was carried out. Various amounts of mte from 0 mg to 12 mg were added to 15 ml falcon tubes along with 5 ml of recombinant mPrP to a final concentration of 0.2 mg/ml. The solutions were stirred for two hours before being centrifuged at 800×g for 10minutes. The supernatant was collected and submitted to BCA analysis. The amount of mPrP remaining in solution was recorded and used to assess the amount that had adsorbed to the mte. Controls that omitted the protein or mte were used. Figure 1 compares the capacity of the clays for the protein. Under the conditions used, mte has an adsorptive capacity of 50 µg of mPrP per mg of mte. The controls excluding the mte or protein prove that no other factors are responsible for the data obtained. Based on the known physical characteristics of the clay used, this gives a specific adsorbance of 150 µg PrP/M^−2^ mte. Kte has an adsorptive capacity of 25 µg of mPrP per mg of kte. This translates to a specific adsorbance of 750 µg PrP/M^−2^ kte. Therefore, 40 mg of both kte and mte was used to adsorb 1 mg of protein for future experiments.

### The Initial Desorption of mPrP from Kte and Mte

Previous reports have suggested that the adsorption of PrP to clay is irreversible [15]. Various desorption conditions were therefore tested to confirm whether desorption was possible. Mte or Kte (40 mg) was added to 1.5 ml eppendorfs along with 1 ml of recombinant mPrP to a final concentration of 1 mg/ml protein. The tubes were then rotated for two hours at room temperature before being centrifuged at 800×g for 10 minutes and the supernatant collected. A variety of solutions were then added to the tubes and thoroughly mixed by vortexing. The tubes were then incubated for 10 minutes at either 25 °C or 100 °C. The solutions used were sodium acetate at pH 3 or pH 5, MES/Tris at pH 8 or pH 10, 10% SDS, Desorption buffer (100 mM Tris-pH 8, 10% SDS, 7.5 mM EDTA, 100 mM DTT, 30% glycerol), 2 M sodium chloride and MilliQ water. After treatment in the solutions, the tubes were allowed to equilibrate to room temperature before again being centrifuged at 800×g and the supernatant collected. The supernatant was then submitted to BCA assay and Western blot analysis. Two antibodies were used, ICMS-18 (anti PrP C-terminal residues 143–153) and 8B4 (anti-PrP N-terminal residues 35–45). Table 1 summarises the attempts at the desorption of mPrP from mte and kte. All methods were more efficient at recovering the protein at 100 °C. By far the most successful method was by boiling the mte in deadsorption buffer, which yielded over 95% recovered protein.

**Table 1:** Summary of the results from the methods used to deadsorb PrP from mte and kte.

Desorption Solution	Temperature (°C)	Amount of mPrP released from mte		Amount of mPrP released from kte	
		mg	%	mg	%
Sodium acetate pH 3	25	0.05	5	0.01	1
Sodium acetate pH 5	25	0.04	4	0.01	1
MES/Tris pH 8	25	0.09	9	0.03	3
MES/Tris pH 10	25	0.09	9	0.05	5
10% SDS	25	0.18	18	0.09	9
Desorption buffer	25	0.39	39	0.20	20
2 M Sodium Chloride	25	0.13	13	0.07	7
MilliQ water	25	0.01	1	n/d	n/d
Sodium acetate pH 3	100	0.14	14	0.11	11
Sodium acetate pH 5	100	0.10	10	0.10	10
MES/Tris pH 8	100	0.11	11	0.09	9
MES/Tris pH 10	100	0.14	14	0.10	10
10% SDS	100	0.28	28	0.24	24
Desorption buffer	100	0.95	95	0.70	70
2 M sodium chloride	100	0.24	24	0.21	21
MilliQ water	100	0.05	5	0.08	8

n/d–not detected.

**Figure 2:**
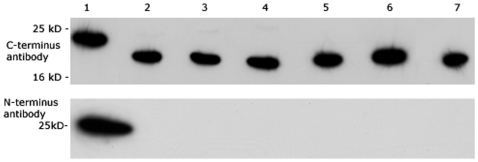
Desorption Causes Protein Truncation. Blot comparing protein desorbed from the clay probed with (i) ICMS-18 and (ii) 8B4 ant-PrP antibody. The conditions are compared with 1) Fresh recombinant PrP and rPrP desorbed from mte with 2) 10% SDS 25°C, 3) desorption buffer 25°C, 4) 2 M NaCl 25°C, 5) 10% SDS 100°C, 6) desorption buffer 100°C, 7) 2 M NaCl 100°C.

When compared to fresh recombinant PrP, the desorbed protein was smaller than expected (Figure 2) by around 2–3 kDa. The antibody used, ICMS-18, is specific to the C-terminus of the protein. An antibody raised to the N-terminus region 35–45 produced no reactivity to protein desorbed by any of the conditions. This suggests that a section of protein at least 20 residues long is missing from PrP post deadsorption. It is not clear at this stage whether this fragment is cleaved by the desorption conditions or if it is left adsorbed to the clay.

### Electrophoretic Desorption as Novel Method for Desorption of Protein from Soils in it Native State

**Figure 3:**
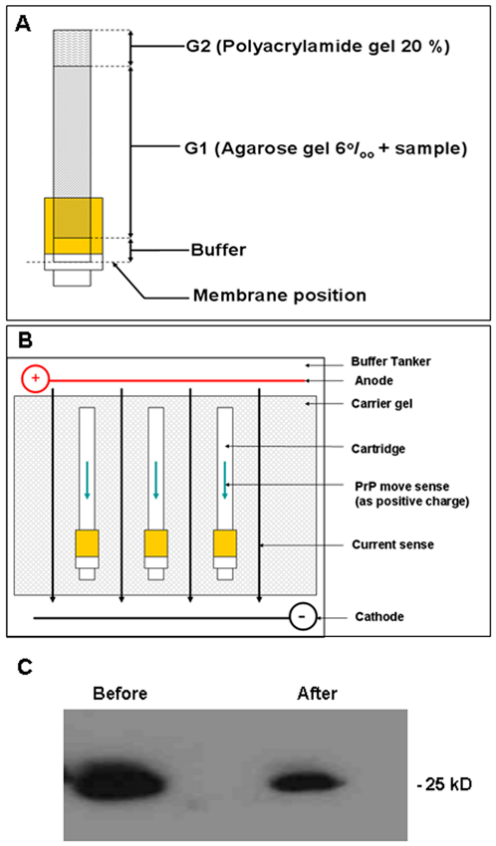
Electrophoretic Desorption. A Schematic diagram of the desorption device. The cartridge with the membrane at the base is loaded with the components as indicated. The agarose component is mixed with the sample and added over the buffer when the cartridge is in the upright position. Once the agarose has solidified the polyacrylamide plug is added. B The cartridge with the components added is laid flat in a standard DNA electrophoresis chamber. Buffer is then added to cover the cartridge and current applied. C Western blot of recombinant PrP detected with and antibody 8B4. The protein was the same molecular weight before and after absorption and desorption from the mte clay matrix and retained its N-terminus.

There is clearly a need to develop a method to remove the adsorbed protein from the clay in a way that will allow for more detailed analysis. A method that does not expose the protein to high temperatures or harsh denaturing conditions was therefore developed. The base idea behind the method is to use the proteins existing electrostatic charge by developing a polar potential difference across the clay, while trapping the substrate and allowing the protein to deadsorb and become trapped on a membrane. PrP is a basic protein with a PI of 9.6. Using this theory, a desorption apparatus was constructed. Protein/clay mixtures in 20 mM MES buffer, pH 5 were set in cooling polyacrylamide gel inside plastic tubes of 5 mm diameter. A 20% polyacrylamide plug was then set into the top of the tube to seal it (Figure 3A). These tubes where then attached to 3 kDa membranes at one end and set into 0.8% agarose gel. The gel was then submerged in a gel tank containing TAE buffer, pH 5 and a current of 35 mV passed across the gel (Figure 3B). The entire apparatus was cooled in ice to prevent overheating. Electrophoretic desorption was tested using 40 mg amounts of recombinant protein/clay mixtures that were electrophoresed for 24 hours. It is clear from figure 3C that the N-terminal region previously lost by the harsh desorption conditions is still present when the protein is extracted by this method. This method of protein desorption was therefore adopted for further studies.

**Figure 4:**
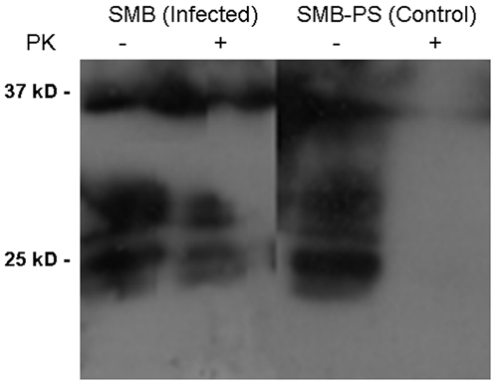
Desorption of Cell Extracts and PK resistance. Western blot and immunodetection for PrP (ICMS-18) of the cell extracts following desorption from mte after 6 months incubation. Desorbed samples were divided in two and half was treated with PK. The results show that the mte retained PK resistant and non resistant protein equivalently.

Extracts were prepared from SMB cells that express proteinase K (PK) resistant PrP and also from SMB-PS that express only PrP^c^, which is PK sensitive. Extracts from the cells were prepared and absorbed onto Mte for six months. Electrophoretic desorption was then used to extract the protein from the Mte and the recovered protein was analysed with western blot (Figure 4). The protein was clearly of the correct size. Adsorption/desorption to the soil matrix had no effect on the PK resistance of the protein as shown by treatment with 8 µg/ml PK for 1 hour at 37°C.

### Effect of Metals on Long Term Survival of PrP on a Soil Matrix

**Figure 5:**
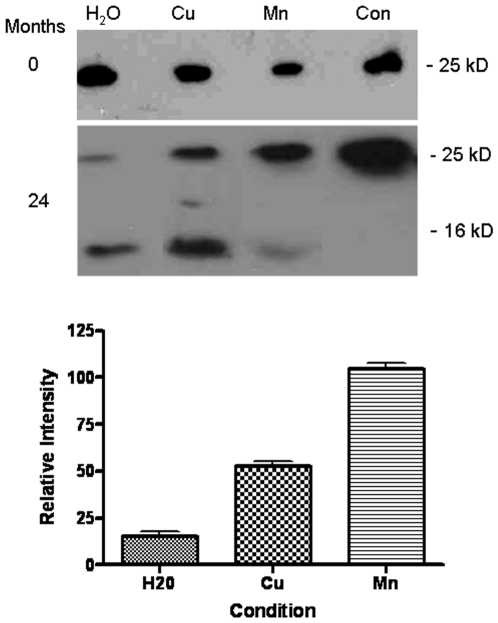
Metals and Survival of recombinant PrP. Western blots to detect PrP (ICMS-18) in recombinant PrP desorbed from mte after either 0 or 24 months. Samples of fresh control protein (con) not absorbed to the mte were also run on the same blots. The mte was either treated with no additional metals (H_2_O) or with the addition of manganese of copper. Densitometric analyses of 3 western blots for each condition is shown at the bottom. Quantitation was of the 25 kD band. All intensity readings are relative to the protein desorbed from the mte with manganese. Shown are the mean and s.e.

In order to investigate whether key metals played any role in the stabilisation of PrP in soil, mte was mixed with copper or manganese. 1 mM solutions of the sulphate salt of each metal were mixed with mte for 2 hours shaking at room temperature. Previous work has shown the adsorpative capacity of mte for Cu and Mn to be relatively equal, with the clay having a capacity of 3.04 mg/g for Cu and 3.22 mg/g for Mn [24]. The mixture was then centrifuged to sediment the soil matrix and the supernatant discarded. The clay was then dried in a dessicator for 72 hours before use. Experiments were also carried out as before to assess the PrP binding capacity of mte after exposure to the metals. No significant differences were observed in the binding capacity of mte and mte-Cu or mte-Mn for rPrP. rPrP solutions of 1 mg/ml were then mixed with 40 mg of mte to produce an aqueous metal/soil/protein mixture and incubated at room temperature for either 0 or 24 months. The protein was then extracted from the clay by the electrophoretic desorption as described above and analysed by western blot and immunodetection for PrP. In each case following desorption, 30 µl of the sample was loaded on the gel. Densitometric analyses was carried out using the Adobe Photoshop package and subjected to ANOVA tests. Figure 5 shows the blot for the protein extracted immediately after 24 hours incubation on the clay. It is clear from figure 5 that, after 2 years incubation on the clay, PrP is able to survive better when in the presence of metals, especially manganese. Relative to the manganese condition, there is around half as much protein recovered from the clay with copper (n = 3, p<0.001) and around 6 times less protein from the clay with no metals present (p<0.001).

**Figure 6:**
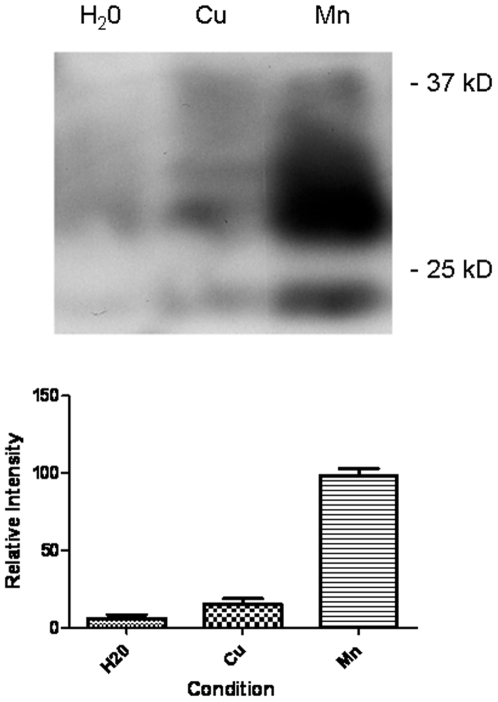
Metals and the survival of native PrP. SMB cell extracts were applied to mte and the residual protein desorbed 24 months later. Western blot and immunodetection for PrP (ICMS-18) were used to detect the relative amounts of PrP that survived the incubation. The mte either contained no additional metals (H_2_O) or copper and manganese. Densitometric analyses of 3 western blots for each condition is shown at the bottom. Quantitation was of the visible bands. All intensity readings are relative to the protein desorbed from the mte with manganese. Shown are the mean and s.e.

In order to verify that this result with recombinant protein was relevant to native protein, the experiment was repeated with protein extracted from SMB cells. Again the protein was absorbed to Mte and left under aqueous conditions for 24 months. After that time the protein was deadsorbed eletrophoretically and the amount of PrP remaining assessed by western blot and immunodetection (Figure 6). The extracts from the aqueous soil with no metals present (lane 1) show only traces of protein remaining. Densitometric analyses reveals only 8±3% protein present relative to the manganese condition (n = 3, p<0.01). The extracts from the aqueous clay in the presence of copper (lane 2) show evidence of an increase in protein stability, but have still suffered significant degradation, with only 16±2% protein relative to the manganese (p<0.001). The extracts from the clay containing the infectious material in the presence of manganese (lane 3) show a considerable amount of PrP still remaining.

### Role of Metal Binding Sites on Protein Survival

**Figure 7:**
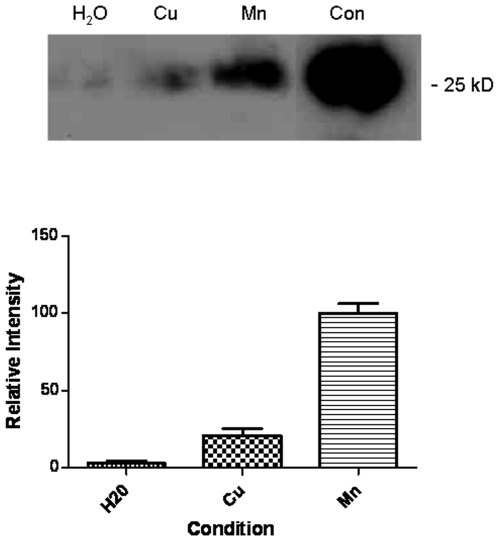
Role of metal binding sites in PrP survival. A recombinant PrP mutant was generated that had the six N-terminal histidines replaced with alanines. This mutant has greatly diminished metal binding capacity. This protein was also absorbed to mte and then desorbed after 24 months. A sample of fresh control protein (con) not absorbed to the mte was also run on the same blot. The mte was either treated with no additional metals (H_2_O) or with the addition of manganese of copper. Densitometric analyses of 3 western blots for each condition is shown at the bottom. Quantitation was of the 25 kD band All intensity readings are relative to the protein desorbed from the mte with manganese. Shown are the mean and s.e.

It is well established that histidines in the N-terminus of PrP are important for the copper binding properties of PrP. We have generated a mutant form of PrP with the six histidines in the N-terminus (four in the octameric repeat region and the histidines at residues 95 and 110) with substitution mutations for alanine. We have previously shown that this mutant does not bind copper [8]. In parallel with wild-type protein, this recombinant PrP mutant protein was absorbed to Mte for two years and then electrophoretic deadsorption was used to recover the protein as described above. After analysis by western blot (Figure 7) it was clear that in comparison to the manganese treated samples, the metal free and copper treated samples were more degraded than the wild-type protein (compare Figure 5). The implication of these findings is that the histidines present in the N-terminus of the protein provide stability for PrP bound to a soil matrix. However, the stability resulting in the interaction with manganese is far greater and is not dependent on the histidines of the N-terminus.

### Manganese and Infectivity

**Figure 8:**
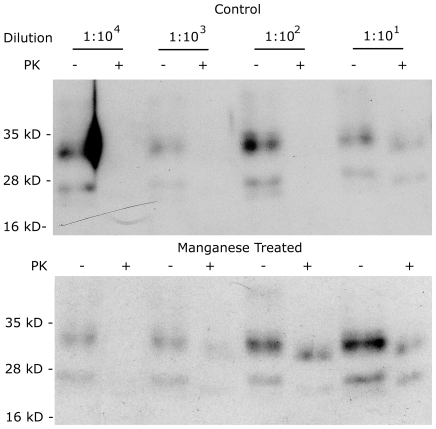
Manganese Enhances Infectivity of Prions. SMB-PS cells, transfected to overexpress mouse PrP, were treated with or without manganese while exposed to PK treated extracts from scrapie infected SMB cells. Different dilutions of the extracts were applied. After treatment and further incubation, protein extracts were prepared from the treated cells. Half of each extract was treated with PK and equivalent amounts electrophoresed and western blotted. Immunodetection of PrP showed bands corresponding to PrP in all PK untreated lanes but bands were only present in PK treated lanes where infection was successful. Treatment with manganese increased the sensitivity of SMB-PS cells to infection. Experiments were repeated 4 times with the same result.

We have demonstrated that manganese present in a model soil can enhance the survival of PrP. We have also previously shown that manganese binds to PrP [9]. What remains to be determined is whether manganese enhances the infectivity of prions. We used a cell cultured based assay system. Uninfected cells (SMB-PS), stably overexpressing mouse PrP via transfection with the plasmid pCDNA3 carrying the open reading frame of mouse PrP, were cultured at 50% confluence and treated with an extract from SMB cells (infected cells) at a range of dilutions from 1∶10 to 1 to 10^4^ . The cells were exposed to the extract for four days after which the cells were washed to remove any residual extract and maintained in culture for a further 7 days. Extracts were prepared from the cells and treated with PK before gel electrophoresis and western blotting to detect PrP. The presence of PK resistant PrP via western was considered indicative of successful infection. Under these conditions infection was only seen at the 1∶10 dilution (Figure 8). Higher dilutions did not result in detectible PK resistant bands. The experiments were repeated in the presence of 50 µM MnSO_4_ in the culture media during the 4 day infection period. Following similar analysis PK resistant bands were also observed for the 1∶10^2^ and 1∶10^3^ dilutions as well as the 1∶10 dilution observed for controls (Figure 8). The implication is that the infectivity of the SMB extract was increase by 100 fold by the presence of manganese. These results demonstrate that manganese enhances prion infection.

Infection experiments were also attempted with Mn-PrP desorbed from the clay matrices. Treatment of the cells resulted in significant cell death. This prevented analysis of the infectivity of the desorbed protein. This was possibly due to trace amounts of the clay matrix remaining associated with the protein.

## Discussion

There is a strong need to establish how TSE's are transmitted from one infected entity to another. Without this knowledge, the full eradication of the disease from both animal and human populations will remain little more than a pipedream. Most evidence suggests that the mode of infection within animals is via the ingestion of infected material [25] and that some of this material is sourced from the environment such as contaminated soil that animals graze [15], [26], [27], [28]. As the TSE's are caused by an infectious protein [6], [29], this evidence raises more questions than it answers, the primary one concerning the proteins mechanism of stability within soil.

There have been many attempts to correlate unusual distributions of soil metals and minerals with incidence of TSE outbreak [16], [30], [31], [32], [33], [34], [35],[36],[37]. These studies have produced a varied group of results, some strongly suggesting that soil mineral and metal contents are important for survival [11], [30], [31], [34], [37], [38], [39] and others suggesting otherwise [32]. Of these studies, many have highlighted imbalances of copper and manganese as possible factors [11], [30], [31], [21], [35]. When this factor is combined with evidence showing these metals have significant affects on protein stability [10] and that infected animals show imbalance of these metals [17], [19], [20], [21], [40] there is clearly a need to assess what affect these metals have on PrP in soil.

The first aim of this study was to assess whether it was possible to remove PrP once adsorbed to the surface of common minerals found in soils. However, normal soils are an unacceptable medium for rigorous scientific analysis because of the multiple variables present in their constituents. The clay montmorillonite is used to provide a controlled substrate for the analysis because of its highly defined character. Initial attempts to remove the protein from the clay provided evidence of just how strong the PrP-mte association is. Despite all the methods tested, it was impossible to remove much protein unless severe denaturing and heat treatments were used. These methods, although effective, meant that any useful information stored within the protein would be lost on desorption. Additionally, significant degradation to the protein was observed which proved to be N-terminal loss, an element not only required for infection but also for stable adsorption to the clay [41]. This fits in well with other tandem studies that have shown PrP is difficult to remove [1], [16], [25], [27] or even impossible [15]. A previous study [16] also demonstrated N-terminal loss on extraction from a clay matrix. None of these methods are therefore suitable for removing protein from the clay matrix.

A previous attempt has been made to extract rPrP from mte using electrophoretic methods [42]. This study relied on harsh denaturing conditions involving SDS and DTT but we wanted to devise an extraction process which would avoid such conditions. Additionally, the study relied on the charge conferred to the protein by SDS binding. All proteins carry a specific charge at pH above or below their point of ionisation (PI). For PrP, a basic PI of 9.6 confers a strong positive charge at neutral or acidic pH. By using this property of protein, PrP was removed successfully from the clay using electrophoretic desorption without any need to denature secondary structure. This allowed for an assessment of how well the entire protein survived on the clay over time. Even in the absence of metals, rPrP resisted degradation remarkable well and after 24 months some trace amounts were still detectible. This supports other studies which have shown increased PrP stability when associated with clays [16], [37]. In fact, these studies have demonstrated that PrP may even be more infectious via oral ingestion when associated with clay. However, a period of around 2 years does appear to reduce the amount of PrP surviving significantly. There was a clear difference between protein adsorbed to clay with metals present and absent. PrP adsorbed to a Cu-mte matrix resisted degradation significantly better than that adsorbed to clay alone, although after 2 years exposure, the majority of the protein had degraded to a 16 kDa fragment. The most striking effect was when protein was adsorbed to a Mn-mte matrix. Even after 24 months, there was little evidence of any significant degradation and the full length protein was still apparent on Western blots. This effect was mirrored by the studies using cellular and disease PrP, an important fact that demonstrated that neither the PK resistance nor glycosylation of the protein play a significant role in the proteins stability in soil. The implication is that the presence of manganese in the soil is the main governing factor as to whether the protein is degraded or not. This observation is closely supported by studies showing that manganese can cause PrP to fold into a protease resistant form [10] and further studies highlighting increased manganese levels in brains of affected individuals [21]. It also provides a potential link to whether studies showing elevated manganese in soil are relevant to disease outbreak [11], [30], [31], [32], [33]. It should be noted, however, that many other factors present in soil would accelerate protein degradation, such as microbial activity. However, given the 10 fold difference in PrP survival in the presence of manganese, if PrP was to survive it is mostly likely to be in a manganese rich soil matrix.

Our laboratory has previously demonstrated some interesting consequences when manganese is bound to PrP, not least the dramatic affects on the protein's redox chemistry [9]. Our work showed that the protein was capable of taking part in and being altered by manganese catalysed redox chemistry that could significantly alter the properties of PrP. This ties in well with other work that demonstrated an affect by manganese oxides on PrP [36], although this study did suggest a deleterious affect, although not in the presence of soil, and with a form of manganese that would be extremely toxic to animals.

The mechanism of PrP stabilisation in the presence of manganese does not appear to involve known metal binding elements within the protein. Studies with Mn-mte using a mutant PrP with all N-terminal histidines removed did not reduce the proteins resistance to degradation when compared to wildtype protein. This suggests that the interaction between Mn and PrP that allows increased survival is via the low affinity site associated with the octa-repeat region in the N-terminus [9] or elsewhere in the protein.

While the ability of the protein to survive in soil is an important aspect of transmission from the environment, infection requires interaction of the protein with cells. Although manganese has been suggested as a risk factor for increased infection, there is currently no evidence that it alters susceptibility to infection. Our data provides evidence for a 100 fold increase in cellular sensitivity to infection in the presence of elevated (non-toxic) manganese levels. These data provide another link in that while manganese may enable prion survival in soil, it would also endow a selective advantage to PrP^Sc^ with manganese bound to cause infection in an animal when ingested. In other words a Mn associated prion is a 100 times more likely to cause infection than a “metal-free” prion. While numerous reports about TSE associations with manganese rich areas have suggested that increased ingestion of Mn leads to increased TSE incidence are possibly misguided in that dietary absorption of manganese is highly limited [43], the possibility that the environment might allow prions to survive and be more infectious is a possibly more reasonable hypothesis.

In conclusion, soil metals do have a significant affect on PrP stabilisation on clays. This is in addition to an already striking ability for the protein to stably adsorb to minerals in soils and survive the harsh conditions in the soil environment. It is therefore possible that where areas of high manganese are present, PrP in both its cellular and disease forms could survive for longer periods and hence potentially be transmitted to new hosts. The association of PrP with manganese potentially makes prions more infectious as cell culture studies show increased susceptibility to infection in the presence of elevated manganese. These results suggest that methods to separate prions from manganese might render them less infectious.

## Methods

Unless stated all materials were purchased from Sigma.

### Model Clays

The two most commonly used model soil systems used for protein-soil interaction studies are montmorillonite (mte) and kaolinite (kte). Mte is a mineral clay of the smectite family and very well defined. Its composition is (Na,Ca)_0.33_(Al,Mg)_2_Si_4_O_10_(OH)_2_ [13]. The Aluminium atoms are situated between two silicon layers sharing the valancies of the oxygens. This forms a tetrahedral structure with an expandable gap between interlayers. This space cannot expand beyond 2 nm [44] and will therefore not allow globular PrP to pass into the clay structure. Kaolinite is another silicate mineral of the phyllosilicate family and is also very well defined, with a composition of Al_2_Si_2_O_5_(OH)_4_ [13]. It is a layered silicate mineral, with one tetrahedral sheet linked through oxygen atoms to one octahedral sheet of alumina octahedral [45]. Again, the gap between sheets does not allow for passage of large globular proteins into the clay.

### Recombinant Protein Generation

Wild-type recombinant mouse prion protein and mutants were prepared as previously described [46]. Briefly, bacterial expression was used to generate recombinant protein. Bacteria were transformed with a plasmid (pET) containing the open reading frame of wild-type mouse PrP (amino acid residues 23–231) or mutants of this construct. Protein expression was induced with 1 mM isopropyl β-D-1-thiogalactopyranoside (IPTG) and inclusion bodies isolated from the bacteria with standard techniques. The inclusion bodies were solubilised in a buffer containing 8 M urea. Recombinant PrP was purified using immobilised metal affinity chromatography (IMAC). The column was charged with copper and the protein bound to the column eluted with 300 mM imidazole in 8 M urea. All proteins were generated tag-free. 0.5 mM EDTA was added to the protein to chelate any metals present. All subsequent steps used double deionised water treated with Chelex resin, to remove residual metal ions. The denatured protein was refolded by a ten fold dilution of the urea in deionised water, concentration by ultra-filtration and two rounds of dialysis to remove residual urea, imidazole and EDTA. Protein concentrations were measured using theoretical extinction coefficients at 280 nm (http://us.expasy.org/tools/protparam.html) and confirmed by BCA assay (Sigma). Protein purity was checked using polyacrylamide gel electrophoresis under denaturing conditions stained with Coomassie brilliant blue.

### Bradford Assay

Triplicate protein standards of 0 to 1 mg/ml bovine serum albumin (BSA) in 0.1 mg/ml intervals were prepared using MilliQ water to 10 µl. For each protein of interest, 10 µl samples were also prepared. Bradford reagent was then diluted 5 fold n MilliQ water. 1 ml of this solution was then added to each of the standards and samples, vortexed and incubated at 25°C for 5 minutes. The solutions were then transferred to plastic cuvettes and spectrographically analysed at 595 nm in a Cary UV Vis spectrophotometer. From the standards, a calibration curve was calculated and used to determine the protein concentration of the samples.

### Western Blotting

Protein samples of either recombinant protein or from cultured cells were electrophoresed on 12% polyacrylamide gels. Protein immobilised within the gel was transferred to a PVDF membrane by a semi-dry transfer method. Blotting paper was soaked in transfer buffer (500 ml 1 x running buffer, 200 ml methanol and 300 ml ddH_2_O). PVDA membrane (pre-soaked in methanol) was placed on top of the blotting paper with the 2D gel placed on top of this. A final piece of blotting paper was placed on top. The transfer was run for 90 minutes at 50 mA, 25 V using a Bio-Rad semi-dry blotter. The membrane was then blocked in tris buffered saline containing 0.1% (v/v) Tween and 5% (w/v) (TBS-T) milk powder for 1 hour, shaking. Membranes were probed with either ICMS-18 or 8B4 monoclonal mPrP primary antibody. After rinsing the membrane in TBS-T 3 times, the antibody was diluted 15000 times in TBS-T containing 5% milk powder overnight at 4°C overnight. The membrane was then washed again in TBS-T 5 times before being incubated to secondary HRP antibody, again diluted 15000 times in TBS-T containing 5% milk powder for 45 minutes at room temperature. Following extensive washing in TBS-T, the membrane was developed for 2 minutes with ECL reagent and exposed to film for between 15 seconds and 30 minutes. A Compact X-ray processor was used to develop the film. The relative amount of protein present on the blot was quantified using the densitometer function in Adobe Photoshop.

### Cell Culture

The cells used to prepare PrP rich extracts were SMB (scrapie mouse brain) which express PrP^Sc^ and the pentosan sulphate cured control line (SMB-PS) which only expresses PrP^c^ [47] were maintained at 37°C and 5% CO_2_ in a humidified incubator. Cells were cultured in DMEM (Lonza) supplemented with 10% FBS (Lonza) in 75 ml culture flasks. Following achievement of 95% confluency, cells were split 1 in 4 to ensure continued health. This was carried out by aspirating off the used media and releasing the cells by digestion with 2 ml trypsin (Lonza) for 5 minutes at room temperature. Trypsin was then inactivated by the addition of 10 ml fresh media and the cells divided into 4 fresh flasks.

For infection experiments SMB-PS cells were plated at 50% confluency in 6 well trays. An extract in PBS was prepared from SMB cells by scraping the cells from the flask. The extracts were sonicated and the debris pelleted by centrifugation at 14 k r.p.m. The protein content of the supernatant was quantified and diluted to 200 mg/ml before digestion with 50 µg/ml proteinase K (PK) for 1 hour at room temperature. The PK activity was inhibited by the addition of pefabloc (Roche). Dilutions of the PK treated extract were then added to the cells for four days. After that time the extract was removed and the washed with fresh medium which was then replaced. The cells were then incubated for a further seven days before the cells were harvested again by scraping the cells from the wells. The cells were extracted in PBS with 1% igepal. Extracts were divided into two halves. Half of which was treated with PK before electrophoresis, western blot and immunodetection for PrP.
